# Maternal curcumin supplementation alleviates intestinal inflammation of *Escherichia coli*-infected offspring via modulating gut microbiome in chickens

**DOI:** 10.1016/j.aninu.2025.04.022

**Published:** 2025-11-29

**Authors:** Yibin Xu, Yujie Huang, Yongwen Zhu, Junyan Wang, Hebatallah K. Elsenousey, Ahmed M. Fouad, Xiajing Lin, Sai Zhang, Yongquan Han, Shijuan Yan, Zongyong Jiang, Shouqun Jiang, Dong Ruan

**Affiliations:** a Institute of Animal Science, Guangdong Academy of Agricultural Sciences, State Key Laboratory of Swine and Poultry Breeding, Key Laboratory of Animal Nutrition and Feed Science in South China, Ministry of Agriculture and Rural Affairs, Guangdong Key Laboratory of Animal Breeding and Nutrition, Guangzhou 510640, China; b Guangdong Provincial Key Laboratory of Animal Nutrition and Regulation, College of Animal Science, South China Agricultural University, Guangzhou 510642, China; c Department of Animal Production, Faculty of Agriculture, Cairo University, Giza 12613, Egypt; d Guangzhou Cohoo Biotechnology Co., Ltd., Guangzhou 510663, China; e Guangdong Key Laboratory for Crop Germplasm Resources Preservation and Utilization, Agro-biological Gene Research Center, Guangdong Academy of Agricultural Sciences, Guangzhou 510640, China

**Keywords:** Maternal curcumin, Offspring chicken, Immunity, Gut microbiome, *Escherichia coli*

## Abstract

The objective of this study was to investigate the effects of maternal curcumin (CUR) supplementation in breeder feed on the growth performance and intestinal health of offspring chickens infected with *Escherichia coli*. A total of 720 peak-laying Qingyuan partridge breeder hens at 28-week-old (initial body weight [IBW] 1950.11 ± 2.54 g) were fed either a basal diet, or a basal diet with 100, 200, or 400 mg/kg CUR for 20 weeks before collecting breeding eggs. After hatching, 360 one-d-old (IBW 34.03 ± 0.18 g) female offspring were divided into five subgroups (72 per each CUR treatment, and 144 for basal diet treatment): offspring of breeders whose mothers were fed the basal diet group (CON); offspring of breeders whose mothers fed a basal diet and challenged with *E. coli* group (EC); and offspring of breeders whose mothers fed the basal diets containing 100, 200, or 400 mg/kg CUR and challenged with *E. coli* group (CUR100E, CUR200E, or CUR400E). All the offspring treatments had 6 replicates of 12 chickens per replicate cage, and chickens bred for 3 weeks. The results indicated that maternal CUR supplementation resulted in a dose-dependent deposition primarily in the yolk. Maternal CUR supplementation (*P* < 0.001) relieved the offspring chickens' weight loss, increased feed conversion rate, and decreased average daily gain, intestinal morphological damage, and goblet cell injury. And maternal CUR supplementation changed in intestinal barrier genes (zonula occludens-1, occludin, and mucin 2), inflammatory factors (tumor necrosis factor α and interleukin-22), and oxidative markers (total antioxidant capacity and glutathione peroxidase) caused by *E. coli* infection (*P* < 0.05). Additionally, maternal CUR supplementation reversed the reduction in offspring chickens’ gut microbial diversity (Shannon and Simpson indexes, *P* < 0.001) caused by *E. coli* infection. Maternal supplementation with CUR demonstrated a significant reduction in the colonization of *Barnesiella* (*P* < 0.001), which was found to have a positive correlation with intestinal inflammatory cytokines and oxidative stress. Concurrently, maternal CUR increased the relative abundance of *Lactobacillus* (*P* < 0.05), which was negatively correlated with intestinal inflammation and oxidative stress. The present study showed that maternal CUR supplementation can alleviate intestinal inflammation in offspring chickens, which may be mediated by regulating gut microbiota composition.

## 1 Introduction

In the poultry industry, the intestines of newly hatched chickens are susceptible to multiple pathogenic factors, including environmental stress, bacterial infection, and mycotoxins, which ultimately result in intestinal inflammation (Abd El-Hack et al., 2022; Wickramasuriya et al., 2022). This inflammation causes severe intestinal mucosal damage, intestinal epithelial dysfunction, and nutrient malabsorption (Barbara et al., 2021; Sylvestre et al., 2023). The intestinal microbiome is often considered as a “virtual organ” within the body and plays a crucial role in protecting the host from intestinal damage (Donald and Finlay, 2023; Wastyk et al., 2021). The intestinal microbiota plays a key role in the maintenance of intestinal mucosal barrier integrity and regulation of host innate and adaptive immunity (Hsu and Schnabl, 2023; Rooks and Garrett, 2016). Thus, it is imperative to investigate novel preventive strategies aimed at mitigating the impact of intestinal inflammation in broiler chickens.

Curcumin (CUR), a natural polyphenol abundant in the rhizome of *Curcuma longa*, has been shown to possess antioxidant, anti-inflammatory, antibacterial, and immunomodulatory properties (Sadeghi et al., 2023). In ducks, quail, and hens, supplemental CUR improved intestinal architecture by enhancing antioxidant status, tight junction function, and immune status under stress-free conditions (Liu et al., 2024; Ruan et al., 2019; Xu et al., 2024b). CUR or bisdemethoxycurcumin exhibited a protective effect on the intestines of broilers and ducks injected with lipopolysaccharide (LPS) (Yang et al., 2023; Zhang et al., 2021). Moreover, our previous research indicated that dietary supplementation of CUR could alleviate LPS-induced enteritis and play an important role in regulating the structure of the intestinal microbiome, promoting health and treating intestinal dysbiosis (Ruan et al., 2022). CUR was deposited in diverse tissues of ducks that were provided with a diet supplemented with CUR, which was in parallel to the corresponding changes in their functions (Ruan et al., 2019). In addition, studies have shown that in fetal growth restriction models, maternal CUR can improve placental function and promote the fetal growth by upregulating the expression of *Nrf2*/*HO-1*, and other antioxidant genes, and reduce the levels of inflammatory factors and oxidative damage indicators (Niu et al., 2019; Qi et al., 2020). Maternal CUR can also alleviate developmental defects of the fetal heart, nerves, and other organs which caused by exposure to harmful substances such as alcohol, arsenic, and mercury (Abu-Taweel, 2019; Poojan et al., 2015; Yan et al., 2017). These findings suggest that CUR could affect mammalian’s offspring health through trans-generational effects. However, it remains unclear whether CUR can deposit in the eggs of egg-type birds or breeders, and affect the immune function of offspring. Studies have revealed that early chicken growth and development depend on the nutrient kept in eggs (Chen et al., 2021; Reicher et al., 2022). The diet of hens can change the egg’s nutritional profile, thus influencing the progeny’s health status (Hafeez et al., 2023; Zhang et al., 2024). On the other hand, changes in the egg’s nutritional profile can also change the intestinal microbiota of chickens (Das et al., 2021; Slawinska et al., 2019). As a natural polyphenol, CUR metabolites such as tetrahydrocurcumin and hexahydrocurcumin exhibit potent antioxidant and anti-inflammatory properties, enabling them to modulate microbial metabolic activity, highlighting the strong connection between CUR and gut microbiota (Pluta et al., 2020; Scazzocchio et al., 2020). Based on these results, it was hypothesized that supplementing CUR in the maternal diet may change the microbial community and affect the immune function of intestines in offspring chickens. It was thus conducted the current study to investigate the trans-generational effects of CUR supplementation in broiler breeders’ diets on offspring chickens.

## 2 Materials and methods

### 2.1 Animal ethics statement

The protocol for the animal experiment was approved by the Animal Welfare and Ethics Committee of the Institute of Animal Science, Guangdong Academy of Agriculture Sciences (No. 2022005).

### 2.2 Diets, animals, and experimental design

A total of 720 peak-laying slow-growing Qingyuan partridge breeder hens (initial body weight [IBW] 1950.11 ± 2.54 g) at 28 weeks old (Zhaoqing Yinongxing Breeding Development Co., Ltd., Zhaoqing, Guangdong, China) were randomly assigned to four treatment groups, each with six replicates of 30 birds. These groups were fed either a basal diet (MCON), or a basal diet with 100, 200, or 400 mg/kg CUR (CUR100, CUR200, or CUR400), respectively. The components of the basal diet are shown in Table 1. During the experiment, the breeders were fed with a restricted intake (110 g/d per pen) at 07:00 and had ad libitum access to water. The treatment of breeders lasted 20 weeks, with artificial insemination conducted during the final two weeks. During the final week of the experiment, 50 eggs were collected from each replicate and incubated. After hatching, the chickens were sexed, and the female chickens were separated. A total of 360 one-d-old (IBW 34.03 ± 0.18 g) female chickens (72 per CUR treatment group and 144 in the control group) were transferred to brooder cages in a room with controlled light and temperature, and bred in the same environmental conditions. The offspring chickens were divided into five subgroups with 6 replicates of 12 chickens per replicate: offspring of chickens whose mothers were fed the basal diet (CON), offspring of chickens whose mothers were fed a basal diet and challenged with *Escherichia coli* (EC), and offspring of chickens whose mothers were fed the basal diets containing 100, 200, or 400 mg/kg CUR and challenged with *E. coli* (CUR100E, CUR200E, or CUR400E). Chickens in the CON group were housed separately from those in the *E. coli* challenge groups to prevent cross-contamination. All offspring chickens were reared in floor pens (3.5 m × 1.2 m) with wood shaving (5-cm deep) and had ad libitum access to feed basal diets and antibiotic-free water. The basal diet of offspring chickens was a corn-wheat-soybean meal-based feed that met the requirements for yellow-feathered chickens according to the NY/T 3645-2020 (Ministry of Agriculture of the People's Republic of China, 2020). Birds were orally administered 2 mL bacterial suspension of *E. coli* suspended in PBS (1 × 10^9^ colony forming unit [CFU]/mL) at 08:00 on d 14, 17, and 20 and were slaughtered on d 21 (Ekim et al., 2020). *E. coli* K88 was obtained from Guangdong Microbial Culture Collection Center (Guangzhou, Guangdong, China). The main active ingredient, CUR, used in the study was extracted from natural turmeric and was provided by Cohoo Biotech Co., Ltd. (Guangzhou, Guangdong, China). Its purity was > 98% by high-performance liquid chromatography (HPLC) analysis. The supplemental dosage of CUR was selected based on previous studies (Ruan et al., 2022).

**Table 1:** Ingredients and nutritional composition of the basal diet for broiler breeders and offspring chickens (DM basis, g/kg).

Ingredients	Content	Composition	Levels
**Broiler breeders**			
Corn	67.30	Metabolizable energy, MJ/kg	11.44
Soybean meal	15.00	Crude protein	16.05
Rapeseed meal	5.00	Calcium	3.01
Corn gluten meal	3.00	Total phosphorus	0.59
L-Lys HCl (98.5%)	0.15	Non-phytate phosphorus	0.37
D,L-Met (99%)	0.13	Total Lys	0.80
L-Thr hydrochloride (98.5%)	0.02	Total Met	0.40
L-Arg (98%)	0.05	Total Met + Cys	0.80
Limestone	6.80	Total Thr	0.63
Dicalcium phosphate	1.25	Total Trp	0.17
Sodium chloride	0.30	Total Ile	0.56
Premix^1^	1.00		
Total	100.00		
**Offspring chicks**			
Corn	52.73	Metabolizable energy, MJ/kg	12.38
Wheat	5.00	Crude protein	21.12
Soybean meal	33.00	Calcium	0.87
Corn gluten meal	3.00	Total phosphorus	0.65
Soybean oil	2.28	Non-phytate phosphorus	0.41
L-Lys HCl (98.5%)	0.06	Total Lys	1.10
D,L-Met (99%)	0.10	Total Met	0.44
Limestone	1.05	Total Met + Cys	0.79
Dicalcium phosphate	1.48	Total Thr	0.74
Sodium chloride	0.30	Total Trp	0.20
Premix^2^	1.00	Total Ile	0.76
Total	100.00		

DM = dry matter.

1 The premix provided per kg of diet: vitamin A, 11,000 IU; vitamin D_3_, 2800 IU; vitamin E, 30 mg; vitamin K_3_, 2.1 mg; vitamin B_1_, 1.1 mg; vitamin B_2_, 9.0 mg; vitamin B_6_, 3.0 mg; vitamin B_12_, 17 mg; choline, 1050 mg; nicotinic acid, 25 mg; pantothenic acid, 3.0 mg; folic acid, 1.1 mg; biotin, 0.11 mg; Fe, 60 mg; Cu, 8.0 mg; Mn, 90 mg; Zn, 80 mg; I, 1.00 mg; Se, 0.12 mg.

2 The premix provided per kg of diet: vitamin A, 12,000 IU; vitamin D_3_, 600 IU; vitamin E, 45 mg; vitamin K_3_, 2.5 mg; vitamin B_1_, 2.4 mg; vitamin B_2_, 5.0 mg; vitamin B_6_, 2.8 mg; vitamin B_12_, 0.01 mg; choline, 1300 mg; nicotinic acid, 42 mg; pantothenic acid, 12 mg; folic acid, 1.00 mg; biotin, 0.12 mg; Fe, 80 mg; Cu, 7.0 mg; Mn, 80 mg; Zn, 85 mg; I, 0.70 mg; Se, 0.15 mg.

Curcumin was extracted from *C.* *longa* L. rhizomes using an ethanol percolation method. Briefly, the rhizomes were crushed to a 30-mesh powder. The powder was pre-soaked in 85% ethanol for 0.5 h, then loaded into a percolator and compacted to remove air pockets. Ethanol was added and the material soaked for 6 h, after which percolation was performed at 3 mL/min until the effluent was nearly colorless. The percolate was concentrated to a paste, subjected to alkaline hydrolysis with 2% sodium hydroxide to remove impurities, and extracted with n-hexane to eliminate lipid-soluble components. Finally, the extract was vacuum-dried, quality-controlled, and packaged for storage.

Nutrient levels in breeder and offspring diets were analyzed by the following methods or calculated according to the requirement of the Yellow-feathered broilers recommended by the NY/T 3645-2020 (Ministry of Agriculture of People’s Republic of China, 2020). Briefly, the crude protein (Kjeltec 8420, FOSS Analytical A/S, Hillerod, Denmark), Ca and total P (AY-BF-888-10, Henan Andy High Temperature Products Co., Ltd., Zhengzhou, Henan, China) were analyzed according to the GB/T 6432-2018, GB/T 6436-2018, and GB/T 6437-2018 (China National Standard, 2018a, China National Standard, 2018b, China National Standard, 2018c).

### 2.3 Samples collection

On d 21, offspring chickens were weighed to evaluate growth performance. Two birds that had average body weight in each replicate were selected. Blood samples collected from the wing vein were allowed to coagulate at 4 °C for 20 min and then centrifuged at 3000 × *g* for 10 min. The serum was subsequently collected and stored at −20 °C. The chickens were electrically stunned and euthanized by exsanguination. The samples of the jejunum and ileum were collected and preserved at −80 °C for gut index and cytokine level analysis. Cecal digesta were also collected and placed in sterile centrifuge tubes and kept at −80 °C for further examination.

### 2.4 Real-time quantitative PCR (RT-qPCR)

The RNA was extracted from jejunal and ileal tissues using the RNAiso Plus kit (9108, Takara Bio Inc., Dalian, Liaoning, China) as per the manufacturer’s instructions. Reverse transcription and RT-qPCR for quantification were conducted according to the procedure described earlier. In short, RT-qPCR was performed using Evo M-MLV RT Premix for qPCR (AG11706, Accurate Biotechnology Co., Ltd., Changsha, Hunan, China). ChamQ Universal SYBR qPCR Master Mix (Q711-03, Vazyme Biotech Co., Ltd., Nanjing, Jiangsu, China) was used for RT-qPCR. The qPCR mixture (10 μL) consisted of 5 μL SYBR Mix, 1 μL cDNA, 0.5 μL of each forward and reverse primer, and 3 μL ddH_2_O. The reaction was performed by the Bio-Rad detection system (CFX96, Bio-Rad Laboratories Inc., Heracles, CA, USA) under the following conditions: 5 min predenaturation at 95 °C, followed by 40 cycles of denaturation at 95 °C in 30 s, annealing at 60 °C in 30 s, and extension at 72 °C in 30 s, and finally extension at 72 °C for 5 min. The primers used were synthesized by the Qingke Biology Co., Ltd. (Guangzhou, Guangdong, China) and are listed in Tables S1 and S2. Each RT-qPCR reaction of each sample was performed in technical triplicates. Primer specificity was validated by melting curve analysis, which showed a single peak for each primer pair. All primer pairs were confirmed to have PCR efficiencies between 95% and 105%, as determined by standard curve analysis using serial dilutions of cDNA. Transcription levels of the genes were measured using the 2^−ΔΔCt^ method. Beta-actin was used as an internal control to standardize the expression of target gene.

### 2.5 Analysis of immune indicators and antioxidant status

The protein concentrations of interleukin (IL)-1β, IL-6, tumor necrosis factor α (TNF-α), and IL-10 (MM-369101, MM-052101, MM-093801, and MM-114501, Jiangsu Meimian Industrial Co., Ltd., Yancheng, Jiangsu, China), and IL-22 (SP37511, Wuhan Saipei Biotechnology Co., Ltd., Wuhan, Hubei, China) were measured with commercially available MEIMIAN ELISA kits designed for chickens in accordance with the instructions provided by the manufacturer. The serum and intestinal activities of glutathione peroxidase (GSH-Px; A005-1-2), total superoxide dismutase (T-SOD; A001-3-2), catalase (CAT; A007-1-1), total antioxidant capacity (T-AOC; A015-1-2), and the content of malondialdehyde (MDA; A003-1-2) in offspring were assessed using a kit from the Nanjing Jiancheng Biotechnology Institute (Nanjing, Jiangsu, China). The absorbance values were detected at a wavelength of 450 nm by using a microplate reader (NEO2S, Bio-Tek Instruments lnc., Winooski, VT, USA). For these cytokines, the intra-assay coefficient of variation were below 8%, and the inter-assay coefficient of variation were below 10%.

### 2.6 Histological analysis

The jejunum and ileum were fixed in 4% paraformaldehyde for 24 h, dehydrated in ethanol, cleared with xylene, and embedded in paraffin. Sections 3-μm thick were then cut and stained with hematoxylin and eosin (H&E). Goblet cell counts were determined using Alcian blue/periodic acid-Schiff (AB-PAS) staining. Images were captured using a light microscope (DMi8, Leica Microsystems GmbH, Wetzlar, Germany). For each group, ten sections were prepared, and six random areas per section were selected for imaging based on tissue type. The measurements of villus height, crypt depth, and goblet cell counts (per 100 μm of villus) were conducted by Image-Pro Plus software (v6.0).

### 2.7 Analysis of egg CUR content by HPLC

The concentration of CUR in both egg white and egg yolk was assessed using HPLC on an Agilent HPLC system (1260, Agilent Technologies Inc., Santa Clara, CA, USA). The analysis was conducted under the following reversed-phase conditions: Phenomenex Gemini C18 (4.6 mm × 250 mm × 5 μm, Phenomenex Inc., Torrance, CA, USA) with the column temperature maintained at 35 °C. Detection was performed at 425 nm using a diode array detector, and the injection volume was 20 μL. The chromatography was carried out using 4% acetic acid (A) and acetonitrile (B) in water as the stationary and mobile phases, respectively. The gradient conditions were as follows: a flow rate of 1 mL/min with 52% A and 48% B for 20 min, followed by 48% B from 20 to 25 min, and then re-equilibration with 52% A and 48% B for 5 min. Chromatograms of CUR were collected and analyzed using the ChemStation software (Agilent Technologies Inc., Santa Clara, CA, USA). The CUR standard (CAS:458-37-7) with a purity of 99.5% was purchased from Sigma–Aldrich Trading Co., Ltd. (Shanghai, China). A total of 6 egg samples per group were randomly selected for HPLC analysis (*n* = 6).

### 2.8 Gut microbiota 16S rRNA sequencing

DNA was extracted from cecal microbiota using the QiaAmp DNA Mini kit (Cat. No. 51306, Qiagen GmbH, Hilden, Germany) following the manufacturer’s protocol. The V3–V4 region of the 16S rRNA gene was amplified via PCR using forward (5′-ACTCCTACGGGAGGCAGCA-3′) and reverse (5′-GGACTACHVGGGTWTCTAAT-3′) primers. Paired-end sequencing was conducted on the Illumina platform (Illumina Inc., San Diego, CA, USA). Primer sequences were removed using Cutadapt (v2.3), and sequences were merged and quality-checked with Vsearch (v2.13.4). Operational taxonomic units (OTU) were clustered at 97% sequence similarity and was performed using Qiime (v2019.4). OTU annotation was performed using the Silva_132 database for species classification and visualized through bar graphs. Selected for its comprehensive coverage of 16S rRNA sequences, regular updates, and alignment-based classification approach, a more specific classification at the generic level may provide a more accurate interpretation of changes in the chicken gut microbiome (Campos et al., 2022). Taxonomic annotations were categorized at the phylum and genus levels. Alpha and β-diversity analyses were performed using Qiime (v2019.4). The Shannon, Chao1, Simpson indexes, and observed_species were applied to assess α-diversity. Beta-diversity was evaluated using normalized OTU abundance tables, including principal coordinate analysis (PCoA) based on Bray–Curtis distances, with significance tested using the analysis of similarities (ANOSIM) method. Linear discriminant analysis (LDA) in combination with effect size (LefSe) was employed to identify differentially abundant taxa and marker bacteria across groups.

### 2.9 Statistical analysis

All data are expressed as means and standard error of the mean (SEM). All analyses were performed using GraphPad Prism (v9.0) and RStudio software (v4.1.3) with the following model:

$$Y_{ijkl}=\mu +\alpha_{i}+\beta_{j}+{\left(\alpha \beta \right)}_{ij}+e_{ijk}\text{,}$$

where *Y*_*ijk*_ is the dependent variable; *μ* is the overall mean; *α*_*i*_ is the effect of *E. coli* challenge; *β*_*j*_ is the dietary treatment effect; (*αβ*)_*ij*_ is the effect due to the interaction between the *E. coli* challenge and the dietary treatment; *e*_*ijk*_ is random error. This mixed-effects model was chosen to account for both fixed effects and their interactions, suitable for this experimental design with multiple factors. The Wilcoxon rank-sum test, as a non-parametric test appropriate for comparing two groups with non-normally distributed microbiome data, was used to determine significance between microbial communities, and Duncan’s multiple comparison analysis was utilized for differences among groups. A Spearman’s correlation analysis was conducted using the corrplot package in RStudio (v2024.04) to assess the correlation between microbiome and inflammatory factors, antioxidant indexes and intestinal barrier-related genes, as is suitable for handling nonlinear relationships and non-normally distributed data. A value of *P* < 0.05 is considered statistically significant, while *P* < 0.01 is regarded as extremely significant.

## 3 Results

### 3.1 Curcumin level in eggs and offspring growth performance

First, the HPLC analysis was performed to examine whether CUR deposition in eggs could be induced through maternal supplementation. The results indicated that CUR was not detected in the MCON group, and CUR was deposited in the egg yolk following maternal supplementation, with significantly higher CUR content in the CUR200 and CUR400 groups compared with the CUR100 group (*P* = 0.003) (Table 2). Subsequently, the feed utilization and growth performance of offspring from CUR-supplemented hens were assessed following *E. coli* infection (Table 3). The results revealed that chickens in the EC group had significantly lower body weight compared with the CON, while chickens in the CUR400E groups exhibited significantly higher body weight than those in the EC group (*P* < 0.001). The average daily feed intake (ADFI) of chickens was lower in the EC group compared to the CON group, although there were no significant differences among groups (*P* = 0.061). The average daily gain (ADG) of EC group was significantly lower than that of CON group, but CUR400 group was significantly higher than EC group (*P* < 0.001). The feed conversion ratio (FCR) of the EC group was significantly higher than that of the CON group, while the CUR400 group was significantly lower than the EC group (*P* < 0.001).

**Table 2:** Effects of maternal curcumin (CUR) supplementation on CUR deposition in eggs (ng/g).

Items	Treatments^1^				SEM	*P*-value
	MCON	CUR100	CUR200	CUR400		
Egg white	ND	18.25	13.89	18.05	2.476	0.744
Egg yolk	ND	85.02^b^	198.20^a^	258.41^a^	23.397	0.003

ND = not detected; SEM = standard error of the mean.

Within a row, means without a common superscript letter differ at *P* < 0.05, *n* = 6 (12 chickens per replicate).

1 MCON, chickens were fed the basal diet; CUR100, CUR200 and CUR400, chickens were fed basal diet supplemented with 100, 200, or 400 mg/kg CUR.

**Table 3:** Effects of maternal curcumin (CUR) supplementation on growth performance of *Escherichia coli*-challenged offspring chickens.

Items	Treatments^1^					SEM	*P*-value
	CON	EC	CUR100E	CUR200E	CUR400E		
IBW, g	34.96	34.79	34.85	36.03	35.32	0.173	0.138
FBW, g	255.83^a^	183.83^c^	193.33^c^	206.83^bc^	218.83^b^	5.049	<0.001
ADFI, g/d	23.05	21.10	22.05	21.89	22.30	0.216	0.061
ADG, g/d	10.52^a^	7.10^c^	7.55^c^	8.13^bc^	8.74^b^	0.240	<0.001
FCR	2.20^c^	3.00^a^	2.94^ab^	2.71^ab^	2.56^bc^	0.067	<0.001
Mortality rate, %	0.90	2.03	0.93	1.23	0.93	0.011	0.341

IBW = initial body weight; FBW = final body weight; ADFI = average daily feed intake; ADG = average daily gain; FCR = feed conversion rate; SEM = standard error of the mean.

Within a row, means without a common superscript letter differ at *P* < 0.05, *n* = 6 (12 chickens per replicate).

1 CON, offspring of chickens whose mothers were fed the basal diet; EC, offspring of chickens whose mothers were fed basal diet and challenged with *E. coli*; CUR100E, CUR200E, and CUR400E, offspring of chickens whose mothers were fed basal diet supplemented with 100, 200, or 400 mg/kg CUR, and challenged with *E. coli*.

### 3.2 Intestinal morphology and barrier-related genes

Intestinal morphology is an essential indicator of intestinal health and resistance to pathogen invasion. The impact of maternal CUR supplementation on the morphology of the jejunum and ileum in *E. coli*-infected chicken offspring was assessed using H&E staining (Table 4 and Fig. S1), and the mRNA expression of intestinal barrier-related genes in the jejunum and ileum was analyzed by RT-qPCR (Table 5). Intestinal morphology data are presented in Table 4. *E. coli* infection significantly shortened villus height and reduced villus height to crypt depth ratio in jejunum and ileum (*P* < 0.05), but it did not significantly affect crypt depth. Compared with the EC group, the CUR400E group significantly increased ileal villus height (*P* < 0.001), and also increased the villus height to crypt depth ratio in jejunum and ileum (*P* < 0.05), while the CUR100E and CUR200E groups did not show significant changes. In addition, *E. coli* infection reduced (*P* < 0.05) the relative mRNA expression of *OCLN*, *ZO-1*, *CLDN1*, *CLDN2*, and *MUC2* genes in jejunum and ileum (Table 5). Compared with the EC group, the mRNA relative expression of *OCLN*, *ZO-1*, and *MUC2* genes of jejunum and ileum was significantly increased in the CUR400E group (*P* < 0.05), while the CUR100E and CUR200E groups did not show significant increases (*P* > 0.05).

**Table 4:** Effects on the intestinal histomorphology and goblet cells of curcumin (CUR) supplementation chicken offspring infected with *Escherichia coli*.

Items	Treatments^1^					SEM	*P-*value
	CON	EC	CUR100E	CUR200E	CUR400E		
**Jejunum**							
Villus height, μm	864.11^a^	792.18^b^	738.29^b^	760.30^b^	790.23^b^	9.700	<0.001
Crypt depth, μm	214.02	217.90	203.69	208.01	199.43	2.483	0.116
Villus height/Crypt depth	4.04^a^	3.60^c^	3.64^bc^	3.69^abc^	3.97^b^	0.046	0.002
Goblet cells, cells/100 μm	9.21^a^	3.58^d^	3.68^cd^	5.30^bc^	5.64^b^	0.356	<0.001
**Ileum**							
Villus height, μm	641.30^a^	561.80^b^	533.38^b^	561.66^b^	624.21^a^	8.785	<0.001
Crypt depth, μm	207.75	222.97	218.25	204.91	210.94	3.762	0.545
Villus height/Crypt depth	3.11^a^	2.51^c^	2.48^c^	2.80^bc^	2.98^b^	0.057	<0.001
Globet cells, cells/100 μm	9.66^a^	4.91^d^	5.12^cd^	5.69^bcd^	6.86^b^	0.325	<0.001

SEM = standard error of the mean.

Within a row, means without a common superscript letter differ at *P* < 0.05, *n* = 6 (12 chickens per replicate).

1 CON, offspring of chickens whose mothers were fed the basal diet; EC, offspring of chickens whose mothers were fed basal diet and challenged with *E. coli*; CUR100E, CUR200E, and CUR400E, offspring of chickens whose mothers were fed basal diet supplemented with 100, 200, or 400 mg/kg CUR, and challenged with *E. coli*.

**Table 5:** Effects of maternal curcumin (CUR) supplementation on inflammatory cytokines, antioxidant indexes and intestinal barrier-related genes of *Escherichia coli*-challenged offspring chickens.

Items^2^	Treatments^1^					SEM	*P*-value
	CON	EC	CUR100E	CUR200E	CUR400E		
**Jejunum**							
**Intestinal barrier-related genes**							
*ZO-1*	1.00^a^	0.39^c^	0.30^c^	0.53^bc^	0.79^ab^	0.053	<0.001
*OLDN*	1.00^a^	0.44^c^	0.48^bc^	0.69^abc^	0.78^ab^	0.059	<0.001
*CLDN1*	1.00^a^	0.49^b^	0.33^b^	0.52^b^	0.45^b^	0.051	<0.001
*CLDN2*	1.00^a^	0.44^b^	0.51^b^	0.44^b^	0.51^b^	0.054	<0.001
*MUC2*	1.00^a^	0.56^c^	0.47^c^	0.69^bc^	0.83^ab^	0.052	<0.001
**Inflammatory cytokines, ng/g**							
IL-1β	38.08^b^	48.00^a^	47.58^a^	44.88^ab^	46.62^a^	0.943	0.002
IL-6	19.59^b^	25.5^a^	24.64^a^	23.74^ab^	23.56^ab^	0.545	0.005
TNF-α	38.95^c^	47.73^a^	49.82^a^	46.31^ab^	42.93^bc^	0.702	<0.001
IL-22	115.39^a^	92.77^c^	95.86^bc^	100.16^bc^	106.32^ab^	1.679	<0.001
IL-10	30.57^a^	22.89^b^	24.95^b^	26.05^b^	25.90^b^	0.539	<0.001
T-SOD	179.07	138.15	138.07	148.41	146.2	5.061	0.055
GSH-Px	247.44^a^	184.09^b^	180.54^b^	216.29^ab^	230.66^a^	6.150	<0.001
CAT	1.88^a^	1.39^b^	1.50^ab^	1.64^ab^	1.59^ab^	0.054	0.048
T-AOC	12.48^a^	9.01^b^	8.99^b^	10.40^ab^	11.43^a^	0.311	<0.001
MDA, nmol/mg	5.68^b^	7.16^ab^	7.64^a^	7.66^a^	7.83^a^	0.225	0.010
**Ileum**							
**Intestinal barrier-related genes**							
*ZO-1*	1.00^a^	0.47^c^	0.48^bc^	0.54^bc^	0.75^ab^	0.042	<0.001
*OLDN*	1.00^a^	0.50^c^	0.61^bc^	0.52^bc^	0.79^ab^	0.051	<0.001
*CLDN1*	1.00^a^	0.41^b^	0.53^b^	0.36^b^	0.59^b^	0.046	<0.001
*CLDN2*	1.00^a^	0.45^b^	0.47^b^	0.54^b^	0.53^b^	0.042	<0.001
*MUC2*	1.00^a^	0.62^c^	0.59^c^	0.70^bc^	0.85^ab^	0.051	<0.001
**Inflammatory cytokines, ng/g**							
IL-1β	36.10^b^	44.96^a^	45.24^a^	44.22^a^	45.86^a^	0.955	0.003
IL-6	17.85^b^	22.73^a^	22.42^a^	21.91^a^	22.73^a^	0.450	<0.001
TNF-α	40.35^c^	49.73^a^	48.29^ab^	47.86^ab^	44.84^bc^	0.676	<0.001
IL-22	104.70^a^	86.16^c^	88.96^bc^	91.96^bc^	96.83^ab^	1.399	<0.001
IL-10	32.78^a^	23.98^b^	22.87^b^	24.69^b^	26.75^b^	0.702	<0.001
**Antioxidant indexes, U/mg**							
T-SOD	207.96^a^	171.06^b^	175.40^ab^	176.87^ab^	192.47^ab^	4.218	0.025
GSH-Px	200.18^a^	146.49^b^	155.24^b^	176.02^ab^	181.87^a^	4.522	<0.001
CAT	1.134	0.87	0.97	0.96	0.99	0.040	0.343
T-AOC	9.76^a^	7.25^c^	6.97^c^	8.32^abc^	9.35^ab^	0.277	<0.001
MDA, nmol/mg	6.25^b^	8.18^a^	8.77^a^	8.40^a^	8.693^a^	0.216	<0.001

IL = interleukin; TNF-α = tumor necrosis factor α; T-SOD = total superoxide dismutase; GSH-Px = glutathione peroxidase; CAT = catalase; T-AOC = total antioxidant capacity; MDA = malondialdehyde; SEM = standard error of the mean.

Within a row, means without a common superscript letter differ at *P* < 0.05, *n* = 6 (12 chickens per replicate).

1 CON, offspring of chickens whose mothers were fed the basal diet; EC, offspring of chickens whose mothers were fed basal diet and challenged with *E. coli*; CUR100E, CUR200E, and CUR400E, offspring of chickens whose mothers were fed basal diet supplemented with 100, 200, or 400 mg/kg CUR, and challenged with *E. coli*.

### 3.3 The density of intestinal goblet cells

The density of goblet cell in jejunum and ileum of offspring chickens was assessed through the utilization of AB-PAS staining. As shown in Fig. S2, maternal CUR supplementation influenced goblet cell density in jejunum and ileum of *E. coli*-challenged offspring. Compared with the CON group, *E. coli* infection significantly reduced the number of both jejunal and ileal goblet cells (*P* < 0.05) (Table 4). However, in comparison with the EC group, the CUR200E and CUR400E groups exhibited a significant increase in jejunal goblet cell density (*P* < 0.001), while the CUR400E group also showed a significant increase in the number of ileal goblet cells (*P* < 0.001). There were no significant changes in the number of jejunal and ileal goblet cells of the CUR100E group (*P* > 0.05).

### 3.4 Intestinal inflammatory response and antioxidant capacity

As shown in Table 5, the effects of maternal CUR supplementation on the inflammatory factors and antioxidant capacities in the intestines of *E. coli*-infected offspring chickens were assessed. *E. coli* infection triggered an inflammatory response in jejunum and ileum, significantly increasing the concentrations of pro-inflammatory cytokines IL-1β, IL-6, and TNF-α, while reducing the levels of anti-inflammatory cytokines IL-22 and IL-10 (*P* < 0.05). In the CUR400E group, the IL-22 concentrations were significantly increased in jejunum and ileum, compared to the EC group (*P* < 0.05), while the concentration of TNF-α was significantly decreased (*P* < 0.05). However, no significant difference were observed in jejunal and ileal IL-1β, IL-6 and IL-10 concentrations, and no significant alterations in inflammatory markers were noted in the CUR100E or CUR200E groups (*P* > 0.05). Additionally, *E. coli* infection decreased the levels of T-AOC and GSH-Px (*P* < 0.05) in both jejunum and ileum, T-SOD in ileum, and CAT in jejunum (*P* < 0.05), while increasing MDA levels in ileum (*P* < 0.001) (Table 5). The activities of jejunal and ileal GSH-Px and T-AOC were increased in CUR400E group (*P* < 0.05), and no significant differences were observed in antioxidant capacity between the CUR100E and CUR200E groups (*P* > 0.05).

### 3.5 Gut microbiota

**Fig. 1:**
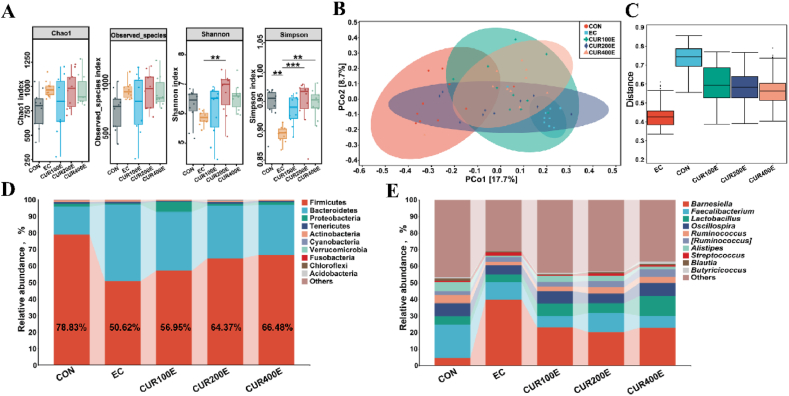
The effects of different maternal curcumin (CUR) supplementation levels on the diversity and composition of the cecal microbiome in offspring chickens infected with *Escherichia coli*. (A) The Chao1, observed_species, Shannon, and Simpson indexes of α-diversity in the different groups. (B) Plots of principal coordinate analysis (PCoA) based on Bray_curtis distance at the genus (C) analysis of similarities (ANOSIM) test based on the Bray_Curtis distance at the genus. (D) Microbial community composition of the cecum at the phylum level. (E) Microbial community composition of the cecum at the genus level. CON, offspring of chickens whose mothers fed the basal diet; EC, offspring of chickens whose mothers were fed basal diet and challenged with *E. coli*; CUR100E, CUR200E, and CUR400E, offspring of chickens whose mothers were fed basal diet supplemented with 100, 200, or 400 mg/kg CUR, and challenged with *E. coli*. ∗∗ *P* < 0.01, ∗∗∗ *P* < 0.001; *n* = 12.

To explore the effects of *E. coli*-infected on the intestinal micrbiome of offspring chickens from the breeders fed CUR, 16S rRNA gene sequencing was performed on cecal digesta of the offspring chickens. The results indicated that the Simpson index (*P* < 0.01) of microbial *α*-diversity was significantly decreased in the EC group compared with the CON group (Fig. 1A), but had no significant effect on the Chao1, observed_species, and Shannon indexes (*P* > 0.05). Compared with the EC group, the CUR200E group significantly increased the Shannon (*P* < 0.01) and Simpson (*P* < 0.001) indexes, and CUR400E groups significantly increased the Simpson index (*P* < 0.01, Fig. 1A). The β-diversity analysis conducted using the PCoA method. PCoA based on Bray_curtis distance showed that the cecal microbiota of the CON group was significantly separated from the EC, CUR100E, CUR200E, and CUR400E groups, and the EC and CUR400E groups were significantly separated (Fig. 1B). Results of ANOSIM further confirmed significant differences (*P* < 0.05) between the EC and CON groups, as well as between the EC and CUR400E groups (Fig. 1C and Table S3). The dominant phyla of chicken cecum microbiome are Firmicutes and Bacteroidetes (Fig. 1D and Table S4), and the relative abundances of the top 10 major genera (*Barnesiella*, *Faecalibacterium*, *Lactobacillus*, etc.) are shown in Fig. 1E and Table S5. The histogram shows that the decreased relative abundance of Firmicutes following *E. coli* infection. In contrast, a higher relative abundance of Firmicutes was observed in the CUR100E, CUR200E, and CUR400E groups and demonstrated a dose-dependent manner (Fig. 1D).

**Fig. 2:**
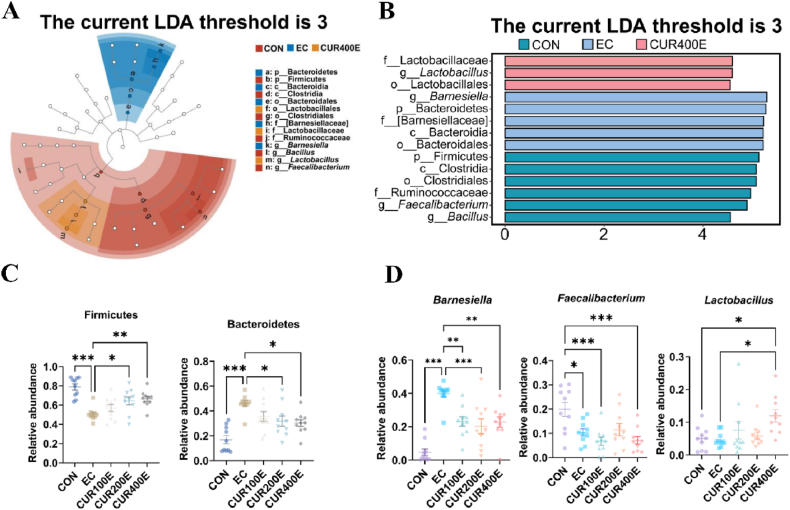
The effects of different maternal curcumin (CUR) supplementation levels on the differences in cecal microbiota of offspring chickens infected with *Escherichia coli*. (A) Cladogram plot of the biomarkers between difference group. (B) Linear discriminant analysis (LDA) of differentially abundant taxa of the cecal microbiota according to effect size (LefSe) between different groups and an LDA score ≥3. (C) The relative abundance of Firmicutes and Bacteroidetes between different groups. (D) The relative abundance of *Barnesiella*, *Faecalibacterium*, and *Lactobacillus* between different groups. CON, offspring of chickens whose mothers fed the basal diet; EC, offspring of chickens whose mothers were fed basal diet and challenged with *E. coli*; CUR100E, CUR200E, and CUR400E, offspring of chickens whose mothers were fed basal diet supplemented with 100, 200, or 400 mg/kg CUR, and challenged with *E. coli*. ∗ *P* < 0.05, ∗∗ *P* < 0.01, ∗∗∗ *P* < 0.001, *n* = 12.

The LefSe results (LDA > 3) showed that the phylum Bacteroides and genus *Barnesiella* were more abundant in the EC group, the phylum Firmicutes and genus *Faecalibacterium* were enriched in the CON group, and genus *Lactobacillus*, as well as the order Lactobacillales and family Lactobacillaceae, were enriched in the CUR400E group (Fig. 2A and B). Differences in the relative abundance of dominant phyla and the top ten genera between the groups were analyzed. Among the top ten genera, only *Barnesiella*, *Faecalibacterium*, and *Lactobacillus* showed significant differences. The infection with *E. coli* significantly decreased the relative abundance of Firmicutes (*P* < 0.001) and increased the relative abundance of Bacteroidetes (*P* < 0.001), while the CUR200E and CUR400 groups showed a significant increase in Firmicutes (*P* < 0.05) and a significant decrease in Bacteroidetes (*P* < 0.05) compared to the EC group (Fig. 2C). The relative abundance of *Barnesiella* (*P* < 0.001) in the EC group was significantly higher than that in the CON group, while the relative abundance of *Barnesiella* in the CUR100E, CUR200E, and CUR400E groups was significantly lower than that in the EC group (*P* < 0.001) (Fig. 2D). The relative abundance of *Faec*a*libacterium* in the EC group (*P* < 0.05), CUR100E group (*P* < 0.001), and CUR400E group (*P* < 0.001) was significantly lower than that in the CON group, and no significant differences were observed on the relative abundance of *Faecalibacterium* among the EC group, CUE100E, CUE200E, and CUR400E groups (Fig. 2D). The relative abundance of *Lactobacillus* in CUR400E was significantly higher than that in the EC group and CON group (*P* < 0.05), and there were no significant differences in the prevalence of *Lactobacillus* between the EC and the CON group (*P* > 0.05; Fig. 2D).

### 3.6 Correlation analysis of intestinal microbial community with inflammatory factors, antioxidant indexes and intestinal barrier-related genes

**Fig. 3:**
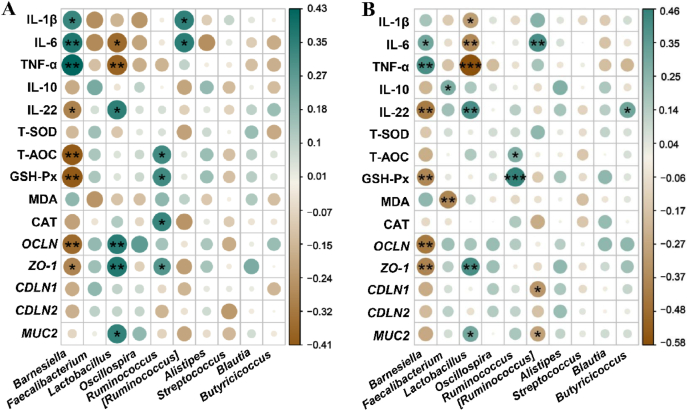
Heatmap of spearman correlation analysis between the genus level of gut microbiota and inflammatory factors, antioxidant indexes and intestinal barrier-related genes. (A) Correlation heat map of jejunum. (B) Correlation heat map of ileum. IL = interleukin; TNF-α = tumor necrosis factor alpha; T-SOD = total superoxide dismutase; GSH-Px = glutathione peroxidase; CAT = catalase; T-AOC = total antioxidant capacity; MDA = malondialdehyde. Brown shading with *P* < 0.05 represents a significant negative correlation, green shading with *P* < 0.05 represents a significant positive correlation, white shading represents no correlation, *n* = 12. ∗ *P* < 0.05; ∗∗ *P* < 0.01; ∗∗∗ *P* < 0.001.

Spearman correlation analyses were conducted to explore potential relationships between the genus level of gut microbiome and inflammatory cytokines, antioxidant markers, and intestinal barrier related genes in jejunum and ileum, as shown in Fig. 3. The relative abundance of *Barnesiella* was positively correlated with the concentrations of IL-1β (*P* < 0.05), IL-6 (*P* < 0.01) and TNF-α (*P* < 0.01), and negatively correlated with the concentration of IL-22 (*P* < 0.05), the activities of GSH-Px (*P* < 0.01), and T-AOC (*P* < 0.01); and the transcript abundance of *OCLN* (*P* < 0.01) and *ZO-1* (*P* < 0.05) in jejunum (Fig. 3A). *Lactobacillus* was positively correlated with the concentrations of IL-6 (*P* < 0.05) and TNF-α (*P* < 0.01), and negatively correlated with the concentrations of IL-22 (*P* < 0.05); the transcript abundance of *OCLN* (*P* < 0.01), *ZO-1*(*P* < 0.01), and *MUC2* (*P* < 0.05) in the jejunum (Fig. 3A). In addition, the relative abundance of *Barnesiella* was positively correlated with the concentrations of IL-6 (*P* < 0.05) and TNF-α (*P* < 0.05), and negatively correlated with the concentrations of IL-22 (*P* < 0.01), the activity of GSH-Px (*P* < 0.01); and the transcript abundance of *OCLN* and *ZO-1* (*P* < 0.01) in ileum (Fig. 3B). *Lactobacillus* was positively correlated with the concentrations of IL-1β (*P* < 0.05), IL-6 (*P* < 0.01), and TNF-α (*P* < 0.001), and negatively correlated with the concentrations of IL-22 (*P* < 0.01); and the transcript abundance of *MUC2* (*P* < 0.01) and *ZO-1* (*P* < 0.05) in ileum (Fig. 3B).

## 4 Discussion

Although numerous studies have explored the immune function impacts of CUR in poultry nutrition (Li et al., 2024b; Liu et al., 2024; Wu et al., 2023a), little is known about its trans-generational impact on offspring. A major characteristic of enteric disease caused by enterotoxigenic *E. coli* infection is epithelial barrier dysfunction induced by the LPS component of its cell wall (Von Mentzer and Svennerholm, 2024). This process activates the inflammatory response, leading to reduced immune function, decreased productive performance, and substantial economic losses in poultry (Ekim et al., 2020; Zhang et al., 2014). This study demonstrated that maternal dietary CUR addition ameliorated intestinal mucosal barrier disruption in chickens exposed to *E. coli* and helped regulate the intestinal microbiota. After 20 weeks of maternal supplementation with varying doses of CUR, CUR accumulated in the yolk in proportion to the dose. Furthermore, maternal CUR supplementation mitigated weight loss, increased FCR and reduced ADG in offspring chickens challenged by *E. coli*. It may be explained that CUR improves lipid metabolism and antioxidant status of embryogenesis, and provides better growth conditions for offspring (Li et al., 2023a). Simultaneously, it increases the level of immunoglobulin in egg yolk, thereby improving the immunity and reducing the negative impact of pathogens in chickens (Gong et al., 2020). Our recent findings indicate that CUR supplementation in feed enhances growth performance, reshapes the intestinal microbiota, and restores intestinal homeostasis disrupted by LPS (Ruan et al., 2022). In the current study, *E. coli* significantly elevated MDA in the ileum compared with the CON, which indicates the presence of stress in this group. In contrast, the jejunal and ileal activities of GSH-Px and T-AOC were increased in CUR400E group compared with the EC group. These results are in line with a previous study in broilers and ducks where adding 150 mg bisdemethoxycurcumin or 500 mg CUR/kg, enhanced GSH-Px and declined MDA in the intestines of LPS-challenged birds. It has been reported that CUR is able to simulate the production of GSH-Px and enhance T-AOC content in the intestines of birds via altering Nrf2 transcript pathways (Wu et al., 2024). Additionally, studies have shown that broilers receiving dietary CUR exhibit improved antioxidant capacity and immune response (Petrone-Garcia et al., 2021; Wu et al., 2023b). Notably, the significant effects of regulating inflammatory markers and enhancing antioxidant capacity observed in the CUR400E group were not significant in the CUR100E and CUR200E groups. This may be due to its low bioavailability and the need for threshold concentrations to exert its effects in offspring tissues. The limited solubility and low bioavailability of CUR suggest that only the highest doses can be sufficiently and subsequently transferred to the offspring intestine and effectively modulate inflammatory and antioxidant responses (Suresh and Nangia, 2018). In addition, the significant changes in TNF-α and IL-22 in the high-dose CUR group, but not IL-1β, IL-6, or IL-10, may be that different cytokines may exhibit varying sensitivities to CUR. TNF-α and IL-22 are key mediators of intestinal inflammation and repair, respectively, and may be more sensitive to the regulation of CUR through pathways such as farnesoid X receptor (FXR) or Nrf2 (Ruan et al., 2022; Wu et al., 2024), while IL-1β, IL-6, and IL-10 may require higher doses or prolonged exposure. These findings suggest that maternal CUR supplementation has significant growth-promoting and intestine protective effects on offspring chickens, and maternal nutrition may have a substantial impact on the offspring’s epigenome.

The gut plays an important role in digestion, nutrient absorption, and host defense. An increase in villus height and a decrease in crypt depth are associated with enhanced nutrient absorption and improved growth performance (Luo et al., 2023; Taylor et al., 2021). Increased villus height and villus height to crypt depth ratio are commonly recognized as indicators of better intestinal epithelial turnover (Felsenthal and Vignjevic, 2022). This turnover is closely linked to intestinal immune function, which is essential for preventing pathogen invasion (Chaukimath et al., 2023). In the present study, *E. coli* infection reduced intestinal epithelial turnover, while maternal supplementation with high doses of CUR improved both intestinal morphology and turnover in chicken offspring. Compared with the EC group, the CUR400E group showed increased ileal villus length and a higher jejunal and ileal villus height to crypt depth ratio, indicating that maternal CUR supplementation positively influenced intestinal morphology and renewal in the offspring. The observed increase in chick weight further supported this improvement.

Maintaining intestinal integrity requires the involvement of tight junctions, which are composed of specific proteins that form a paracellular permeability barrier and prevent the translocation of macromolecules (Balda and Matter, 2023; Paradis et al., 2021). Following *E. coli* infection, intestinal tight junctions were disrupted, as indicated by decreased mRNA expression of *OCLN*, *ZO-1*, *CLDN1*, and *CLDN2* in the jejunum and ileum. This finding aligns with previous studies. Reduced expression of these tight junction proteins is associated with increased intestinal permeability and barrier disruption, allowing macromolecules to pass the bloodstream from the lumen of the intestine (Lin et al., 2024). However, compared with the EC group, the jejunal and ileal mRNA expression of *OCLN* and *ZO-1* in the CUR400E group was significantly increased. Tight junction proteins OCLN and ZO-1 are closely linked to intestinal permeability and integrity (Haas et al., 2022). The elevated mRNA expression of *OCLN* and *ZO-1* in the CUR400E group suggests that maternal CUR supplementation mat enhance the integrity of the intestine damaged by *E. coli* infection. Notably, these significant improvements in intestinal morphology and gene expression were exclusive to the highest dose, with lower doses (100 and 200 mg/kg) showing no significant effects. This pattern suggests a dose-dependent response, potentially driven by CUR’s low bioavailability, where only the 400 mg/kg dose achieved a sufficient concentration in egg yolk to protect the intestinal barrier, consistent with studies indicating higher CUR doses are needed for significant physiological effects in poultry (Liu et al., 2024). Future pharmacokinetic studies could clarify the optimal dosage by tracing CUR’s transfer from breeder hens to offspring.

The maintenance of the gut mucosal barrier relies on the production of mucus (Yao et al., 2022). Goblet cells primarily synthesize and secrete this mucus, which forms a protective layer over the intestinal epithelium, serving as a frontline defense against microbes, pathogens, and environmental toxins (Gustafsson and Johansson, 2022). Protein MUC2 is the principal mucin responsible for maintaining the intestinal mucus gel layer, and its expression is regarded as a marker of intestinal health (Nystrom et al., 2021). Although mucins are continuously synthesized and released at basic levels and can mediate mucosal homeostasis, the changes in the composition and thickness of the mucus layer can rapidly affect the response to pathogens or alterations in the microbiome, regulated by innate and adaptive immune responses. The results demonstrated that *E. coli* infection adversely affected intestinal morphology and significantly reduced the number of jejunal and ileal goblet cells. However, in the CUR400E group, the number of goblet cells in these regions was significantly higher compared to the EC group, indicating increased mucus production that forms a protective mucus layer and reinforces the intestinal barrier against *E. coli*-induced damage. This could reduce pathogen adhesion and improve resistance to intestinal infection. The increased goblet cell density may support nutrient absorption and growth performance by maintaining a healthy intestinal environment, as evidenced by the improved body weight outcomes in these groups. Overall, the findings suggest that maternal CUR supplementation protects gut goblet cells from *E. coli*-induced damage. Enhanced intestinal integrity may also bolster defenses against pathogenic invasion to safeguard goblet cells from harm.

There is an interplay between intestinal immune function and the microbiota, which is important in maintaining intestinal structure and function (McCallum and Tropini, 2024). Cecal microbiota changes indicate gut nonspecific immune responses (Forssten et al., 2023). Infection with pathogens can disrupt gut microbiome homeostasis (Liang et al., 2024; Xu et al., 2024a). The use of 16S rRNA sequencing to analyze gut microbiota composition has been widely accepted, but some taxa may be missed or quantified inaccurately due to primer specificity and inability to resolve the strain capacity of the microbiota, and 16S rRNA sequencing mainly provides taxonomic resolution at the generic level (Bars-Cortina et al., 2023). In results, the α-diversity mainly assesses microbial diversity and richness within a single sample, where the Chao1 and observed_species indexes are often used to estimate microbial richness, the Shannon and Simpson indexes are used to measure microbial diversity (Xu et al., 2022). The α-diversity results showed that *E. coli* infection significantly reduced microbial diversity in the cecum. Results of PCoA and ANOSIM identified significant differences in β-diversity among the groups, indicating that EC and CUR treatments obviously affected cecal microbiota structure. Bacterial infections may cause the dysbiosis of intestinal flora, and maternal CUR supplementation could reverse the imbalance of intestinal flora by enhancing intestinal antioxidant and anti-inflammatory activities. At the phylum level, *E. coli*-infected chickens showed a decreased proportion of Firmicutes and an increased proportion of Bacteroidetes. Interestingly, chickens from breeders fed CUR showed the opposite trend. As a major bacterial phylum, Firmicutes play an important role in nutrient absorption and short-chain fatty acids (SCFA) production (Li et al., 2024c; Xu et al., 2022). Increased Firmicutes abundance improves intestinal barrier function and inhibits inflammatory responses (Li et al., 2024a). These results suggest that maternal CUR supplementation not only regulates the gut microbiota composition of offspring but also enhances their intestinal health and immune function by increasing the proportion of Firmicutes. At the genus level, *E. coli* infection increased the proportion of *Barnesiella*, while maternal CUR supplementation reduced its abundance. The prevalence of *Barnesiella* is positively associated with pro-inflammatory factors and inversely associated with antioxidants. This aligns with studies showing that *Barnesiella* is elevated in the gut of HIV-infected individuals, associated with systemic inflammation, and positively correlated with the pro-inflammatory factor TNF-α (Dinh et al., 2015). Previous studies have shown that *Barnesiella* is an important member of the microbiome of depression patients, which is often accompanied by reduced antioxidant capacity (Zheng et al., 2021), which is similar to this study findings. Furthermore, *Barnesiella* abundance has been found to increase following oxidative DNA damage from ionizing radiation, indicating its potential role in modulating immune responses (Maier et al., 2014). Its abundance is also linked to immunomodulatory cells, including B cells in the bursa of Fabricius and natural killer T cells in the liver and spleen (Presley et al., 2010). The proportions of *Lactobacilales*, Lactobacillaceae, and *Lactobacillus* were higher in the CUR400E group compared with the other groups. The proportion of *Lactobacillus* in CUR400E and CUR200E groups were considerably higher than those in the EC group. Although *E. coli* infection did not directly alter *Lactobacillus* abundance, CUR supplementation in the maternal diet may promote *Lactobacillus* proliferation in the offspring’s intestines. *Lactobacillus*, a beneficial bacterium, plays an essential role in promoting intestinal health, regulating immune function, and maintaining microbial homeostasis (Zhang et al., 2023). The increase in *Lactobacillus* abundance is linked to reduced intestinal inflammation. *Lactobacillus* may causally reduce inflammation via SCFA production and immune cell modulation, as supported by its negative correlation with pro-inflammatory cytokines and positively correlated with antioxidants (Jiang et al., 2023; Li et al., 2023b; Wang et al., 2023a). *Lactobacillus* can inhibit the expression of TNF-α through the nuclear factor kappa-B (NF-κB) pathway to reduce the gut inflammatory response (Haque et al., 2024). In addition, *Lactobacillus* improves the intestinal barrier and reduces intestinal inflammation by activating the native macrophages into the mannose receptor (CD206) macrophages and release IL-10 (Jia et al., 2022), which is in accordance with the findings in this study. Maternal CUR supplementation was shown to alleviate the reduction in IL-22 induced by *E. coli*. The relative abundance of *Lactobacillus* is known to positively correlate with IL-22 levels, a cytokine mainly secreted by type 3 innate lymphoid cells and is crucial for the regeneration of intestinal epithelial cells (Wang et al., 2023b). Oral *Lactobacillus* supplementation triggers the IL-17-dependent innate immune response, activates type 3 innate lymphocytes, and improves colitis (Hrdý et al., 2022). Therefore, maternal CUR supplementation may reduce intestinal inflammation caused by *E. coli* by increasing *Lactobacillus* abundance in the offspring’s intestines. The mechanisms by which maternal CUR supplementation alters gut microbiota remain unclear. Previous research suggests that the chicken embryo microbiota is partially inherited from the maternal cloaca, vagina and oviduct and may be influenced by host genetic variation (Diez-Méndez et al., 2023; Yang et al., 2017). Given that dietary CUR supplementation affects the gut microbiota of chickens, it was hypothesized that maternal supplementation of CUR alters the microbiota composition of offspring, thereby affecting their intestinal immune function. Additionally, nutrients in egg yolk may influence the gut microbiota of chickens (Bełdowska et al., 2024; Dunisławska et al., 2024). As a polyphenolic compound, CUR may interact bidirectionally with gut microbiota, acting as a prebiotic to promote the development of beneficial microbiota (Jabczyk et al., 2021; Zhao and Jiang, 2021)—such as *Lactobacillus*—and reducing opportunistic pathogen colonization. Polyphenols, due to their phenolic structure, also exhibit strong antioxidant properties to scavenge free radicals, which may protect intestinal tissues (Lippolis et al., 2023; Shen et al., 2022). This mechanism may explain how maternal CUR supplementation alleviates *E. coli*-induced intestinal oxidative stress in offspring. Moreover, maternal CUR supplementation may induce epigenetic changes in offspring, such as DNA methylation, histone modification, or micro-RNA expression, potentially affecting genes related to immunity and metabolism (Hassan et al., 2019).

This study revealed the trans-generational effects of maternal CUR on the intestinal health and growth of offspring chickens. However, there are some limitations. The research only used female Qingyuan partridge chicken as a model and a single *E. coli* strain for infection. In addition, the epigenetic mechanism of the trans-generational effects of CUR has not yet been elucidated. Further research should include sex, breed, and test multiple pathogens such as *Salmonella* to investigate epigenetic mechanisms, like DNA methylation, which could reveal how CUR works and optimizing its use in poultry breeds.

## 5 Conclusion

In summary, the present study revealed that maternal 400 mg/kg CUR supplementation could relieve intestinal inflammation by increasing body weight, ADG, antioxidant status, tight junctions, and goblet cell secretion, reducing feed conversion rate and inflammatory cytokines, and regulating gut microbiota structure in offspring chickens challenged with *E. coli*. Maternal supplementation with CUR may contribute to the prevention of early-age intestinal damage in chickens.

## Credit Author Statement

**Yibin Xu:** Writing – original draft, Conceptualization, Formal analysis, Methodology, Data curation. **Yujie Huang:** Formal analysis, Software, Validation. **Yongwen Zhu:** Data curation, Validation. **Junyan Wang:** Validation, Data curation. **Hebatallah K. Elsenousey:** Data curation. **Ahmed M. Fouad:** Validation, Data curation. **Xiajing Lin:** Validation, Data curation. **Sai Zhang:** Data curation, Validation. **Yongquan Han:** Supervision. **Shijuan Yan:** Supervision. **Zongyong Jiang:** Supervision. **Shouqun Jiang:** Supervision, Project administration. **Dong Ruan:** Conceptualization, Methodology, Funding acquisition.

## Availability of data and materials

The 16S rRNA sequence dataset supporting the conclusions of this article is available in the Genome Sequence Archive repository (https://ngdc.cncb.ac.cn/gsa; GSA: CRA019535).

## Declaration of competing interest

The authors declare the following financial interests/personal relationships which may be considered as potential competing interests: Yongquan Hand is currently employed by Guangzhou Cohoo Biotechnology Co. Zongyong Jiang is an Editorial Board Member for Animal Nutrition and was not involved the editorial review or the decision to publish this article.
