# Supplementary material for: Maternal curcumin supplementation alleviates intestinal inflammation of Escherichia coli-infected offspring via modulating gut microbiome in chickens
Source: Anim Nutr. 2025 Nov 29;24:344–55. doi: 10.1016/j.aninu.2025.04.022 (PMC12914816; doi:10.1016/j.aninu.2025.04.022)
Supplement: Multimedia component 1 [file mmc1.docx]

**Table S1** Abbreviations of genes with corresponding full names.

| Abbreviations | Full names |
| --- | --- |
| *FXR* | Farnesoid X receptor |
| *HO-1* | Heme oxygenase-1 |
| *MUC2* | Mucin 2 |
| *Nrf2* | Nuclear factor erythroid 2-related factor 2 |
| *OCLN* | Occludin |
| *ZO-1* | Zonula occludens-1 |

**Table S2** Primer sequences used for real-time quantitative PCR.

| Genes | Direction | Primer sequence (5′-3′) | NCBI references |
| --- | --- | --- | --- |
| *OCLN* | Forward  Reverse | TCATCCTGCTCTGCCTCATCT  CATCCGCCACGTTCTTCAC | NM_205128.1 |
| *ZO-1* | Forward  Reverse | CCAAAGACAGCAGGAGGAGA  TGGCTAGTTTCTCTCGTGCA | XM_015278981.1 |
| *CLDN1* | Forward  Reverse | GAGGATGACCAGGTCAAGAAG  TGCCCAGCCAATGAAGAG | NM_001013611.2 |
| *CLDN2* | Forward  Reverse | TGAACCATTCGCAGTCCCTG  GGGAGGAGAGGTTACAGAGAT | NM_001277622.1 |
| *MUC2* | Forward  Reverse | CATTCAACGAGGAGAGCTGC  TTCCTTGCAGCAGGAACAAC | XM_046942297.1 |
| β-Actin | Forward  Reverse | GAGAAATTGTGCGTGACATCA  CCTGAACCTCTCATTGCCA | NM_205518 |

NCBI = National Center for Biotechnology Information.

**Table S3** Statistical results of analysis of similarities (ANOSIM) among groups^1^.

| Group1 | Group2 | Sample size | Permutations | *R* | *P*-value | *Q*-value |
| --- | --- | --- | --- | --- | --- | --- |
| All | - | 50 | 999 | 0.241 | 0.001 | - |
| CON | EC | 20 | 999 | 0.560 | 0.001 | 0.002 |
| CON | CUR100E | 20 | 999 | 0.335 | 0.001 | 0.002 |
| CON | CUR200E | 20 | 999 | 0.223 | 0.007 | 0.010 |
| CON | CUR400E | 20 | 999 | 0.300 | 0.005 | 0.008 |
| EC | CUR100E | 20 | 999 | 0.279 | 0.001 | 0.002 |
| EC | CUR200E | 20 | 999 | 0.258 | 0.001 | 0.002 |
| EC | CUR400E | 20 | 999 | 0.408 | 0.001 | 0.002 |
| CUR100E | CUR200E | 20 | 999 | 0.015 | 0.353 | 0.353 |
| CUR100E | CUR400E | 20 | 999 | 0.054 | 0.123 | 0.137 |
| CUR200E | CUR400E | 20 | 999 | 0.116 | 0.035 | 0.044 |

CUR = curcumin; - = not detected.

^1^CON, offspring of chickens whose mothers were fed the basal diet; EC, offspring of chickens whose mothers were fed basal diet and challenged with *E. coli*; CUR100E, CUR200E and CUR400E, offspring of chickens whose mothers were fed basal diet supplemented with 100, 200, or 400 mg/kg CUR, and challenged with *E. coli*.

**Table S4** The relative abundance of the top 10 phyla (%) among groups.

| Phyla | Treatments^1^ |  |  |  |  |
| --- | --- | --- | --- | --- | --- |
|  | CON | EC | CUR100E | CUR200E | CUR400E |
| Firmicutes | 78.83 | 50.62 | 56.95 | 64.37 | 66.48 |
| Bacteroidetes | 16.87 | 46.20 | 35.51 | 31.83 | 30.28 |
| Proteobacteria | 1.71 | 0.86 | 6.10 | 1.15 | 1.38 |
| Tenericutes | 1.07 | 1.01 | 0.45 | 1.18 | 1.00 |
| Actinobacteria | 1.13 | 0.90 | 0.52 | 0.69 | 0.51 |
| Cyanobacteria | 0.12 | 0.02 | 0.06 | 0.06 | 0.02 |
| Verrucomicrobia | 0.01 | 0.02 | 0.04 | 0.17 | 0.02 |
| Fusobacteria | 0.00 | 0.01 | 0.00 | 0.01 | 0.00 |
| Chloroflexi | 0.00 | 0.00 | 0.01 | 0.00 | 0.00 |
| Acidobacteria | 0.00 | 0.00 | 0.00 | 0.00 | 0.00 |
| Others | 0.27 | 0.35 | 0.35 | 0.52 | 0.28 |

CUR = curcumin.

^1^CON, offspring of chickens whose mothers were fed the basal diet; EC, offspring of chickens whose mothers were fed basal diet and challenged with *E. coli*; CUR100E, CUR200E and CUR400E, offspring of chickens whose mothers were fed basal diet supplemented with 100, 200, or 400 mg/kg CUR, and challenged with *E. coli*.

**Table S5** The relative abundance of the top 10 genera (%) among groups.

| Genus | Treatments^1^ | |  |  |  |
| --- | --- | --- | --- | --- | --- |
|  | CON | EC | CUR100E | CUR200E | CUR400E |
| *Barnesiella* | 4.67 | 39.96 | 23.18 | 20.26 | 22.88 |
| *Faecalibacterium* | 19.91 | 10.4 | 6.72 | 11.5 | 7.14 |
| *Lactobacillus* | 5.07 | 4.69 | 7.58 | 5.85 | 11.98 |
| *Oscillospira* | 8.04 | 5.52 | 7.52 | 5.85 | 7.88 |
| Ruminococcus | 4.98 | 2.14 | 2.76 | 3.91 | 3.78 |
| *Ruminococcus* | 2.38 | 2.86 | 2.81 | 3.67 | 4.42 |
| *Alistipes* | 5.48 | 0.83 | 3.74 | 3.25 | 1.77 |
| *Streptococcus* | 0.74 | 2.02 | 0.4 | 1.43 | 0.81 |
| *Blautia* | 1.47 | 0.46 | 0.44 | 0.73 | 0.74 |
| *Butyricicoccus* | 0.69 | 0.24 | 0.84 | 0.37 | 1.39 |
| Others | 46.57 | 30.87 | 44.01 | 43.19 | 37.22 |

CUR = curcumin.

^1^CON, offspring of chickens whose mothers were fed the basal diet; EC, offspring of chickens whose mothers were fed basal diet and challenged with *E. coli*; CUR100E, CUR200E and CUR400E, offspring of chickens whose mothers were fed basal diet supplemented with 100, 200, or 400 mg/kg CUR, and challenged with *E. coli*.


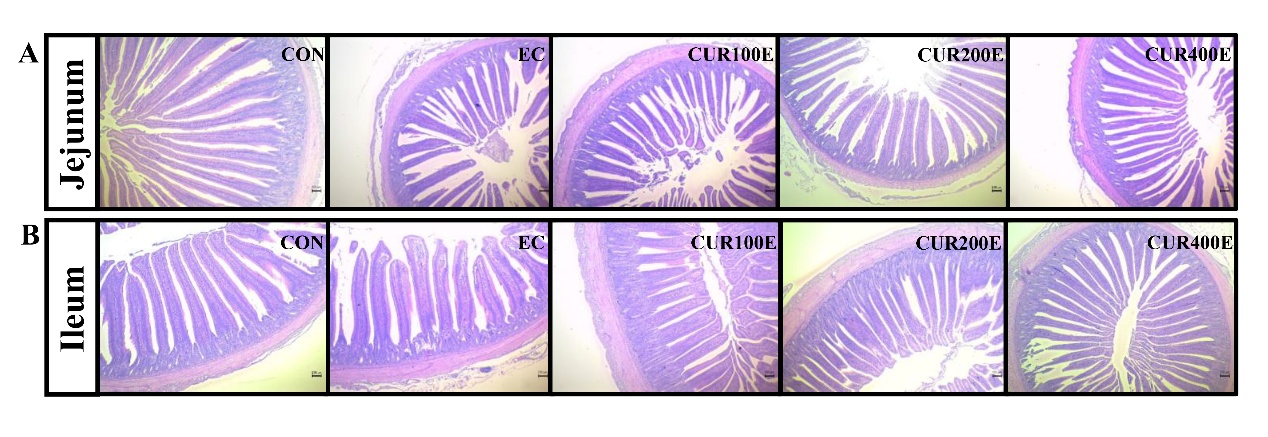


Fig. S1 The effects of different maternal curcumin supplementation levels on the intestinal barrier of offspring chickens infected with *Escherichia coli*. Hematoxylin and eosin (H&E) staining on the jejunum(A) and the ileum (B). The scale bar (200 µm) is located in the lower-right corner. CON, offspring of chickens whose mothers were fed the basal diet; EC, offspring of chickens whose mothers were fed basal diet and challenged with *E. coli*; CUR100E, CUR200E and CUR400E, offspring of chickens whose mothers were fed basal diet supplemented with 100, 200, or 400 mg/kg CUR, and challenged with *E. coli*.


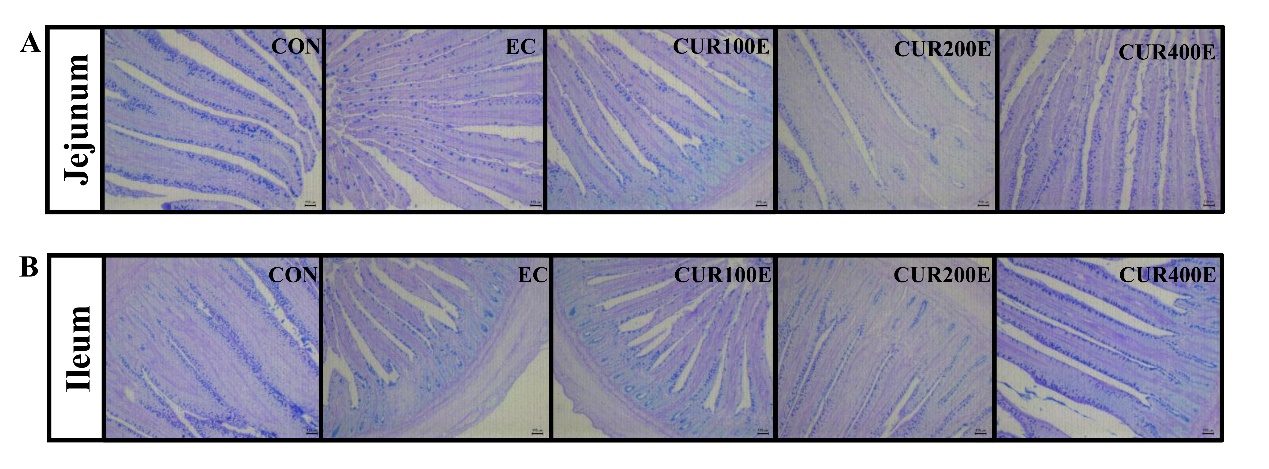
Fig. S2 The effects of different maternal curcumin supplementation levels on the intestinal goblet cells of offspring chickens infected with *Escherichia coli*. Alcian blue/periodic acid-Schiff (AB-PAS) staining on the jejunum (A) and the ileum (B). The scale bar (200 µm) is located in the lower-right corner. CON, offspring of chickens whose mothers were fed the basal diet; EC, offspring of chickens whose mothers were fed basal diet and challenged with *E. coli*; CUR100E, CUR200E and CUR400E, offspring of chickens whose mothers were fed basal diet supplemented with 100, 200, or 400 mg/kg CUR, and challenged with *E. coli*.
